# Self-management processes, sedentary behavior, physical activity and dietary self-management behaviors: impact on muscle outcomes in continuing care retirement community residents

**DOI:** 10.1186/s12877-021-02691-z

**Published:** 2022-01-12

**Authors:** Murad H. Taani, Scott J. Strath, Rachel Schiffman, Michael Fendrich, Amy Harley, Chi C. Cho, Yosuke Yamada, Christine R. Kovach

**Affiliations:** 1 College of Nursing, University of Wisconsin–Milwaukee, 1921 East Hartford Avenue, Milwaukee, WI 53211 USA; 2 College of Health Sciences, University of Wisconsin–Milwaukee, 2400 E Hartford Ave, Milwaukee, WI 53211 USA; 3 School of Social Work, University of Connecticut, Mansfield, USA; 4 UWM Joseph J. Zilber School of Public Health, University of Wisconsin–Milwaukee, 1240 N 10th St, Milwaukee, WI 53205 USA; 5 National Institute of Health and Nutrition, National Institutes of Biomedical Innovation, Health and Nutrition, Tokyo, 162-8636 Japan

**Keywords:** Older adults, Physical function, Protein intake, Caloric intake

## Abstract

**Background:**

Despite the known benefits of non-sedentary behavior, physical activity, and protein and caloric intake to health and muscle mass, strength, and function, many older adults do not meet physical activity and dietary recommendations. A better understanding of the factors associated with sedentary behavior, physical activity and dietary self-management behaviors, and muscle outcomes (muscle mass, strength, and function) is needed, particularly among continuing care retirement community residents. The objective of this study was to examine the factors associated with sedentary behavior, physical activity and dietary self-management behaviors, and muscle outcomes among continuing care retirement community residents. It also aimed to determine whether sedentary behavior and physical activity and dietary self-management behaviors mediate the relationships between self-efficacy, goal congruence, aging expectations, social support, and muscle outcomes.

**Methods:**

A sample of 105 continuing care retirement community residents (age > 70 years) participated in this correlational, cross-sectional study. Questionnaires on pain, self-efficacy, goal congruence, aging expectation, social support, and daily protein and caloric intake were administered. Physical activity and sedentary behavior (ActiGraph wGT3X-BT), muscle mass (ImpediMed SFB7), muscle strength (Jamar Smart Digital Hand Dynamometer), and muscle function (Short Physical Performance Battery) were measured. Multiple regression, logistic regression, and mediation analyses were performed.

**Results:**

Low goal congruence predicted engagement in sedentary behavior and light physical activity. Higher levels of self-efficacy and social support were associated with increased likelihoods of achieving greater moderate physical activity and meeting daily recommendations for caloric intake, respectively. Self-efficacy and goal congruence predicted muscle function and strength. Moreover, sedentary behavior and achieving greater moderate physical activity were found to partially but significantly mediate the relationship between self-efficacy and muscle function.

**Conclusion:**

Future research should evaluate whether attempts to reduce sedentary behavior and promote physical activity and dietary self-management behaviors and muscle outcomes are more successful when modifications to the self-management process factors are also targeted.

## Background

Sarcopenia (i.e., the loss of muscle mass, strength, and function) is associated with negative outcomes including falls, fractures, disability, institutionalization, and mortality [1, 2], and is amplified in sedentary and malnourished older adults. Physical activity and adequate dietary intake, specifically protein and calories, can mitigate declines in muscle mass, strength, and function among older adults [2, 3]. Yet, older adults have life challenges and several other factors that can influence their sedentary behavior and physical activity and dietary self-management behaviors (SMBs).

Despite the known benefits of physical activity to health and muscle outcomes in aging, the total sitting time increased among older adults population between 2007 to 2016 [4] and only 27% of older adults meet recommended physical activity guidelines [5]. Older adults also consume far less protein and calories than the daily recommended amounts for health [3, 6, 7]. This is due, in part, to the physical activity and dietary SMBs and their antecedent factors being overlooked in older adults, particularly older adults living in continuing care retirement communities (CCRCs) [8]. Better understanding of the factors associated with sedentary behavior, physical activity and dietary SMBs, and muscle outcomes among CCRC residents lays the foundation for designing interventions to improve muscle mass, strength, and function and prevent sarcopenia.

**Fig. 1:**
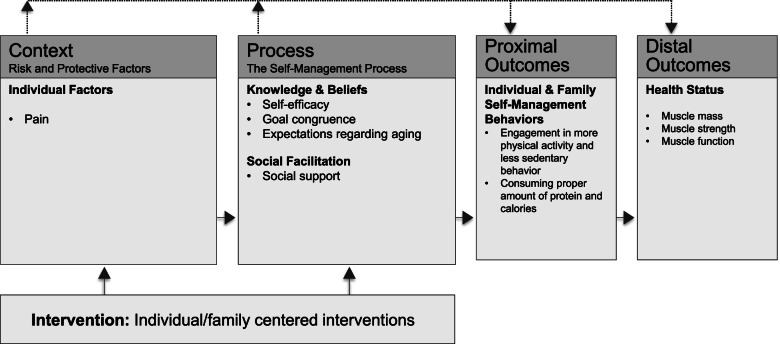
Individual and Family Self-Management Theory Applied to Physical Activity and Dietary Self-management in Older Adults. Adapted with permission from Ryan and Sawin (2009)

This study was guided by the Individual and Family Self-Management Theory [9] (Fig. 1). The study postulated that pain as an individual factor and self-efficacy, goal congruence, aging expectations, and social support as self-management process factors impact sedentary behavior and physical activity and dietary SMBs which in turn impact muscle mass, strength, and function (Fig. 1). Prior research suggested that since pain intensity negatively interfered with self-management and contributes to the progression of sarcopenia [10, 11]. The lack of self-efficacy is also considered a barrier to effective SMBs [12, 13]. Goal congruence describes the degree to which the goals of the individual can be met—for this study, relative to physical activity and dietary SMBs. When individuals can meet their goals regarding their health condition, this leads to better SMBs and outcomes [12]. Aging expectations play an important role in the adoption of a healthy lifestyle and influence health-promoting behaviors such as sedentary behavior, physical activity and a healthy diet [14, 15]. Social support may facilitate self-management by enhancing an individual’s motivation to engage in SMBs [16].

This study was undertaken to: 1) examine the relationship of pain intensity, self-efficacy for physical activity, goal congruence, aging expectations, and social support to sedentary behavior and physical activity and dietary SMBs; 2) examine the relationship of pain intensity, self-efficacy for physical activity, goal congruence, aging expectations, and social support to muscle mass, strength, function; and 3) evaluate whether sedentary behavior and physical activity and dietary SMBs mediate the relationships between self-efficacy, goal congruence, aging expectations, social support, and muscle mass, strength, and function among CCRC residents.

## Methods

### Design, setting, and sample

A cross-sectional correlational design using a convenience sample was utilized for this study. A sample of 105 older adults were recruited from 6 CCRCs in a mid-western city in the United States between October 2018 and June 2019. The CCRCs were selected by convenience sampling. CCRCs are residential housing for retirement older adults that provide various service to maintain an independent lifestyle. The continuum of facilities and services available to residents includes independent apartments, housekeeping, social and recreational activities, a limited amount of on-site medical care, and some transportation. Inclusion criteria were: English speaking, age 70 and older, have a score > 26 on the Montreal Cognitive Assessment (MoCA) [17], and have a score < 11 on the 15-item Geriatric Depression Scale (GDS) [18]. Exclusion criteria included those older adults who were unable to stand without assistance (using assisted devices such as canes or walkers was allowed), who have medical conditions that would limit the ability to increase protein intake such as kidney disease, who have cognitive impairment or depressive symptoms according to MoCA and GDS scales, respectively. A priori power analysis was conducted with 80% power to detect a medium effect size with a 0.10 significance level with five independent variables and two mediation variables included in a model; employing Short Physical Performance Battery (SPPB) as the primary outcome variable. The power analysis indicated that a minimum sample of 85 participants was needed, and 105 participants were recruited. The study was approved by the institutional review board of the University of Wisconsin Milwaukee and written consent was obtained from the participants.

### Measurement

*Pain intensity* was assessed using the Patient Reported Outcome Measurement Information System (PROMIS) Scale Pain Intensity 3a [19]. The scale consists of three items with two questions asking about the intensity of pain in the past 7 days at its worse and on average and one question regarding the intensity of the pain at the present moment. The PROMIS scores are weighted T-score calibrated so scores for each patient based on 50 + 10 being the mean SD of samples derived from U.S. population [20].

*Self-efficacy for physical activity* was assessed by the Physical Activity Assessment Inventory [21]. The scale consists of 13 items that ask participants to rate how confident they feel to increase their usual physical activity in various circumstances. The responses for each item range from 0 (cannot do at all) to 100 (certainly can do). The sum score ranges between 0 and 1300, with higher scores indicating a higher level of self-efficacy. Cronbach’s alpha was .95, and content validity was supported in previous studies [21, 22].

*Goal congruence* was measured using an instrument created by and adapted with the permission of the developers [23]. The original tool focused on physical activity and calcium intake for osteoporosis treatment, but it was modified to focus on physical activity and protein and caloric intake. Multiple professionals with appropriate expertise verify usefulness of the modified instrument. The instrument has 19 questions with a Likert scale from 1 (no problem) to 5 (significant struggle or problem), with a score range of 19 to 95. A high score means low goal congruence. The preface to all questions on the goal congruence instrument was “How much of a problem would it be for you to ….” Topics in the instrument included such issues as “Figure out how to increase your protein intake” and “Feel safe when you engage in physical activity.” This tool measures the individual’s goals related to their physical activity and protein and caloric intake, and whether the individual has the desire and ability to meet those goals. The response categories rate “how much of a problem” achieving each goal is; accordingly, high scores indicate more problems and less congruence. Cronbach’s alpha in our sample was 0.83.

*Aging expectations* was assessed by the Expectations Regarding Aging (ERA-38) survey [24]. The survey includes 38 questions related to several domains including general health, physical and mental health, cognitive function, sleep, and fatigue. The ERA-38 was designed to measure the extent to which individuals expect to experience age associated decline. This instrument has demonstrated acceptable reliability and validity in a previous field test among older adults as a measure of expectations regarding aging [24]. Higher scores indicate a higher expectation to achieve successful aging, and lower scores indicate expected decline in the domains such as physical and mental decline. The total scores were used in the analysis.

#### Social support

The amount of positive social support participants reported receiving from their families, friends, and health and fitness experts was assessed using the Social Influence Scale (SIS) [25]. The scale consists of two subscales: positive and negative social influences. The occurrence of social influences over the last 12 months is measured on a 5-point scale (from 0 = never to 4 = very often). The 15 questions on positive family support are summed and divided by 15 to give the total score, and a higher score indicates greater social support. Evidence of content validity and reliability (Cronbach’s alpha 0.67–0.91) has been shown [25].

*Physical activity and sedentary behavior* were measured objectively by the Actigraph GT3X+ accelerometer (Actigraph, Inc., FL, USA). This accelerometer is a small (3.8 × 3.7 × 1.8 cm) light weight device (27 g) that was worn at the level of the hip and attached to an elastic belt worn around the waist. The device was initialized to record data second-by-second and was worn for all waking hours for 7 consecutive days. We used standardized data quality procedures to assess validity of the accelerometer data and create categories of activity intensity based on accelerometer counts [26, 27]. Briefly, upon retrieval and download via Actilife software 6.13.4, data equal to or > 60 min where the accelerometer activity count data was zero was considered time when the monitor was not worn and was subsequently removed from analysis. To be considered a valid day of accelerometer wear, there had to be a minimum of 600 min of wear time per day. Although all participants were asked to wear the device during all waking hours for 7 consecutive days, some did not wear it for the full time or had days when they had < 600 min of usable data. Only participants who had at least 4 days of valid accelerometer data were included for final analysis. Each minute of accelerometer data was coded as sedentary activities (0 to 100 counts), light physical activity (LPA, 101 to 1951 counts), moderate physical activity (MPA, 1952 to 5924 counts), or vigorous physical activity (VPA, > 5925 counts) [28, 29]. The Actigraph accelerometer has been shown to be a valid and reliable activity monitor across many different populations [29, 30].

*Daily dietary protein and caloric intake* were assessed using the 70-item Block Brief food frequency questionnaires (FFQ) [31, 32]. Daily intake of protein adjusted for body weight was calculated, and a dichotomous variable characterizing protein intake was set: meets recommendation or does not meet recommendation [33]. Daily intake of calories adjusted for age, sex, and physical activity level was calculated, and a dichotomous variable characterizing protein intake was created as follows: 1 = meets recommendation; 0 = does not meet recommendation [34].

### Outcome variables

*Muscle mass* was measured using a tetrapolar bioelectrical impedance spectroscopy (BIS) device. The details of BIS have previously been described [8, 35]. Briefly, BIS is a simple, safe, and noninvasive method for estimating body composition (i.e., body fat and muscle mass). The BIS device sends a weak electric current through the body and the voltage is measured in order to calculate impedance (resistance) of the body. Measurements were taken between the right wrist and ankle with subject in a supine position. The Sergi equation [35] was used to calculate appendicular skeletal muscle mass (ASMM) as recommended by the European Working Group on Sarcopenia in Older People [2].

#### Muscle strength

Handgrip strength was measured using a Jamar Smart Digital Hand Dynamometer® (Jamar Technology Inc., USA) [36]. Participants used their dominant hand unless otherwise instructed and three trials were performed with remained seated with their arm resting on the chair arm as they squeezed the dynamometer. The mean score of the 3-test trials performed was analyzed.

*Muscle function* was measured using the SPPB test [37]. The test includes measures of standing balance, 4-m gait speed, and time needed to rise from a chair five times. For balance, participants were asked to remain standing with their feet side by side, followed by the semi-tandem (heel of one foot along side the big toe of the other foot) and tandem position (heel of one foot directly in front of and touching the other foot) positions for 10 s each. Gait speed was assessed by asking participants to walk at their usual pace over a 4-m course. Two trials were performed and recorded, the faster of the two was used to compute the gait speed score. For the ability to raise from a chair, participants were asked to stand up and sit down 5 times as quickly as possible with arms crossed over their chests. This was done only after participants first demonstrated the ability to rise once without using their arms. The total SPPB score was analyzed.

### Procedure

Flyers were distributed and announcements in the facilities bulletin were placed. The study PI/RA set up an information table in each setting to enhance recruitment. Following consent, a screening form was administered and if the individual was found to be eligible, two visits were scheduled, one and half hours each and 7 days apart, to take place at the CCRC. The two visits were scheduled for convenient times for each participant. During the first visit, demographic questions, pain intensity, Physical Activity Assessment Inventory, and goal congruence measures and the FFQ were administered in an interview format. The SPPB and handgrip strength tests were performed by each participant. All data were collected by a research assistant and the principal investigator. To ensure safety, participants wore a gait belt and were accompanied by two research staff members during the SPPB test. Participants wore an accelerometer on an elasticated belt around the waist, placed on the right hip in line with the right kneecap over the course of a 7 consecutive day period according to instructions and written materials provided. Participants were asked to wear the monitor during all waking hours, except when bathing, swimming, or doing any other water-based activity, and to complete the wear time diary for the same period of 7 consecutive days.

Participants were contacted via phone at day 1, 2, 4, and 6 after the first visit only to check if they were experiencing any discomfort related to wearing the accelerometer. The phone calls did not include any guidance or encouragement to physical activity. In the second visit, accelerometers were collected, and SIS and ERA-38 questionnaires were administered. Strategies to reduce burden and retain participants included contacting participants via phone 4 times after the first visit to check if they were experiencing any discomfort related to wearing the accelerometer, offering breaks during assessment visits to minimize burden, and making every effort to complete study procedures at convenient times.

### Data analysis

Descriptive statistics were used to describe the study variables. MPA data were not normally distributed. The distribution showed two major groups: one group with 0–3 min per day of MPA and a second group with more than 3 min per day of MPA based on median split. Therefore, participants were dichotomized into high MPA (> 3 min per day) and low MPA (0–3 min per day). Bivariate analysis was performed using independent samples t-test and chi-square test of association for continuous and categorical variables, respectively. To address Aim 1, multiple regression models were used to examine factors associated with sedentary behavior and LPA. Logistic regression models were employed to examine factors associated with the dichotomous outcomes (MPA > 3 min per day; and meeting recommendations for protein and caloric intake). For Aim 2, multiple regression models were used to examine factors associated with SPPB, muscle mass, and handgrip strength. Stepwise model selection with entry and removal criteria of *p* = .05 was used to find the best set of covariates for each outcome of interest after controlling for age and sex. In addition to the covariates used in Aim 1, physical activity measures and protein and caloric intake recommendations were also considered for the models in Aim 2. To address Aim 3, mediation analysis was performed using R statistical software and the mediation package following the steps described by Baron and Kenny [38] and using bootstrap estimation for statistical testing. An alpha level of 0.05 was used to evaluate significant model effects; non-significant interaction effects were excluded from the final model. Cases (*n* = 6) with missing data were also excluded from analysis and data of 99 participants were used in the analyses. All non-mediation-related analyses were performed using SAS 9.4 (SAS Institute, Inc., Cary, NC).

## Results

Table 1 shows the characteristics of participants. Among the 105 participants, the majority were female, White, with a mean age of 82.4 years. The participants spent approximately 75 and 21% of their day engaging in sedentary and LPA, respectively. Only 42.9 and 32.4% of the participants met the recommendations for protein and caloric intake, respectively.

**Table 1:** Baseline Characteristics (*N* = 105)

Characteristics	Mean (SD)	n (%)	Range
Age	82.4 (7.4)		70–99
Gender			
Male		19 (18.1)	
Female		86 (81.9)	
Race			
White		87 (82.9)	
African American		16 (15.2)	
American Indian or Alaska Native		2 (1.9)	
Pain Intensity	43.4 (8.8)		30.7–67.4
Self-Efficacy	915.5 (256.6)		220–1300
Goal Congruence	35.9 (11.7)		19–78
ERA	40.3 (15.2)		2.6–93.9
Social support	3.4 (2.6)		0–11.6
Caloric intake			
Meet recommendation		34 (32.4)	
Do not meet recommendation		71 (67.6)	
Protein intake			
Meet recommendation		45 (42.86)	
Do not meet recommendation		60 (57.14)	
Sedentary behavior %	0.75 (0.08)		0.56–0.92
LPA %	0.21 (0.06)		0.06–0.37
MPA %			
0–3 Minutes per Day	73 (73.74)		
> 3 Minutes per Day	26 (26.26)		
ASMM (kg)	16.04 (3.64)		9.79–26.34
Male	20.97 (2.56)		17.9–26.34
Female	14.99 (2.90)		9.79–23.21
Grip Strength (kg)	18.73 (7.07)		2.30–39.07
Male	27.41 (7.14)		15.67–39.07
Female	16.91 (5.57)		2.3–31.97
SPPB	7.89 (2.74)		1–12

Sedentary behavior %: proportion of daily time (out of 100%) spent in sedentary behavior from accelerometer counts less than 100 per minute. LPA %: proportion of daily time (out of 100%) spent in light physical activity from accelerometer counts 100–1952 per minute. MPA %: determined by accelerometer counts greater than 1952 per minute, dichotomized into those achieving less than or greater than 3 min per day

*ERA* Expectations regarding aging, *LPA* Light physical activity, *MPA* Moderate physical activity, *ASMM* Appendicular skeletal muscle mass, *SPPB* Short physical performance battery

### Factors associated with physical activity and dietary SMBs

In the models for physical activity and sedentary behavior, the results showed that higher goal congruence scores (indicative of low goal congruence) were significantly associated with increased sedentary behavior (β = 0.281) and reduced LPA (β = − 0.29) (Table 2). In the logistic regression for MPA, higher self-efficacy was significantly associated with increase odds of achieving at least 3 min of MPA (Odds Ratio (OR) = 1.003; 95% Confidence Interval (CI) 1.001–1.005) (Table 3). The logistic regression results showed that higher ERA scores were significantly associated with a reduction in the odds of meeting the daily recommendation for protein (OR = 0.96; 95% CI 0.93–0.99) and caloric intake (OR = 0.96; 95% CI 0.92–0.99). The results also showed that higher levels of social support were significantly associated with greater odds of meeting the daily recommendation for caloric intake (OR = 1.24; 95% CI 1.04–1.47) (Table 3). The goodness-of-fit statistic (*p* > .05) for this model was acceptable.

**Table 2:** Multiple Regression Models Predicting Sedentary Behavior, LPA, Muscle Strength, and Muscle Function

Predictors	% Sedentary		% LPA		Grip strength		SPPB	
	β	***P***	β	***P***	β	***P***	β	***P***
Self-Efficacy					0.196	0.018	0.358	<.001
Goal Congruence	0.281	0.004	−0.290	0.003	−0.199	0.015	−0.199	0.049
*R-Square*	0.175		0.176		0.507		0.236	
*F (P-value)*	F(3, 95) = 6.73 (*p* = .0004)		F(3, 95) = 6.78 (*p* = .0003)		F(4, 99) = 25.41 (*p* < .0001)		F(4, 100) = 7.71 (*p* < .0001)	

*LPA* Light physical activity, *SPPB* Short physical performance battery

**Table 3:** Logistic Regressions Predicting SM behaviors of Protein Intake, Caloric Intake, and MPA

Predictors	Protein				Caloric				% MPA			
	EST	SE	OR (95% CI)	***P***	EST	SE	OR (95% CI)	***P***	EST	SE	OR (95% CI)	***P***
Age	0.03	0.03	1.03 (0.94–1.09)	0.303	− 0.07	0.03	0.93 (0.87–0.99)	0.026	−0.09	0.04	0.91 (0.85–0.98)	0.015
Gender Female	1.58	0.64	4.88 (1.38–17.19)	0.013	1.43	0.70	4.16 (1.06–16.30)	0.040	−0.76	0.61	0.47 (0.14–1.53)	0.206
Expectation Regarding Aging	−0.04	0.02	0.96 (0.93–0.99)	0.008	−0.05	0.02	0.96 (0.92–0.99)	0.012				
Social Support					0.21	0.09	1.24 (1.04–1.47)	0.017				
Self-efficacy									0.003	0.001	1.003 (1.001–1.005)	0.0060

*MPA* Moderate physical activity

### Factors predicting muscle mass, strength, and function

The multiple regression models showed that higher levels of self-efficacy were significantly associated with higher handgrip strength (β = 0.196) and SPPB (β = 0.358) scores. Higher goal congruence scores, indicating low goal congruence, were significantly associated with decreased handgrip strength (β = − 0.199) and SPPB (β = − 0.199) scores. However, for muscle mass, after controlling for the significant effects of age and gender, none of the individual self-management process variables were found to be significantly associated with muscle mass.

### Mediation effects

In the analysis examining whether meeting daily dietary recommendation for protein and caloric intake and sedentary behavior and physical activity mediated the relationship between self-management process variables and muscle outcomes, significant mediation effects were found only for the muscle function (SPPB) outcome. Only the percent time sedentary and achieving at least 3 min of MPA were found to significantly mediate the effects of self-efficacy on SPPB. Specifically, the percent time spent sedentary was found to mediate 17.6% of the effect of self-efficacy on SPPB (*p* < 0.001) and achieving greater minutes of MPA mediated 22.2% of the effect of self-efficacy on SPPB (*p* = 0.02) (Table 4).

**Table 4:** Analysis of Mediator Effect for Muscle Function (SPPB)

		Mediator					
		% Sedentary			MPA		
Direct Effect	Measure	EST	95% CI	***P***	EST	95% CI	***P***
Self-Efficacy	ACME	0.001	(0.00, 0.00)	<.0001	0.001	(0.00, 0.00)	0.02
	ADE	0.004	(0.00, 0.01)	<.0001	0.004	(0.00, 0.01)	<.0001
	Total Effect	0.005	(0.00, 0.01)	<.0001	0.005	(0.00, 0.01)	<.0001
	Proportion Mediated	0.176	(0.04, 0.33)	<.0001	0.222	(0.07, 0.45)	0.02

*ACME* Average causal mediation effect, *ADE* Average direct effect

## Discussion

Our findings indicate that our sample spent approximately 75% of their day engaging in sedentary activity and only 21% of their day in LPA. Only 26.26% of the participants engaged in more than 3 min of MPA per day. We found that 42.9 and 32.4% of the residents consumed the recommended amount of protein and calories, respectively. These findings are consistent with other studies in CCRC residents which reported that most of their samples spent the majority of the day in sedentary activity [39, 40]. Other studies have shown that poor appetite and altered satiety responses are highly prevalent and associated with reduced daily intake of nutrients and malnutrition among community-dwelling older adults and long-term care residents [41, 42]. Older adults intake of protein and calories is far less than the Recommended Dietary Allowance (RDA) [3, 6, 7] and only 30% of adults aged 50 years and older meet the RDA (0.8 g protein/day/kg body weight) for daily protein intake [41]. The combined effects of physical inactivity and malnutrition, particularly inadequate protein and caloric intake, may be particularly deleterious and can accelerate the loss of muscle mass, strength, and function; contribute to the development and progression of sarcopenia; and increase risk of mortality [2, 43, 44]. The findings from our study and other studies should prompt effective efforts to promote physical activity and dietary SMBs among CCRC residents. Healthy aging requires good self-management skills and participation in regular physical activity and healthy eating [45].

We found that CCRC residents with low goal congruence had a significantly higher level of sedentary behavior and lower level of LPA. To our knowledge, this has not previously been reported in older adults. We also found those with higher self-efficacy had an increased likelihood of achieving at least 3 min of MPA per day. These findings are consistent with the existing literature that supports the importance of goal congruence and self-efficacy in promoting SMBs among community-dwelling older adults [12, 13, 23]. The findings also suggest that the self-management process variables of goal congruence and self-efficacy should be a core component of intervention research to reduce sedentary behavior and promote physical activity SMB in CCRC residents.

Our study showed CCRC residents with higher aging expectations had a decreased likelihood of consuming the recommended amounts of protein and calories. This finding is contradictory to the findings of other studies that demonstrated older adults who expected fewer age-related health problems (i.e., had higher ERA) engaged in more routine health-promoting behaviors [14, 15, 46]. Older adults who attribute illness to old age report less routine health-promoting behaviors and are less likely to engage in behaviors such as exercising, eating a healthy nutritious diet, getting a regular check-up, and avoiding a state of inaction (i.e., not ignoring one’s health problems) [46]. Older adults in our sample with positive aging expectations may not be aware of the role of adequate protein and calories for maintaining physical function. We also found older adults with higher positive social support were more likely to consume the recommended amount of calories, which is consistent with prior studies that reported social support facilitates self-management by enhancing one’s motivation to engage in SMBs such as eating healthy diet [16, 47]. Future intervention should focus on improving social support and older adults’ aging expectations in conjunction with an emphasis on their active involvement in health-promoting behavior.

Our sample of CCRC residents showed poor muscle mass, strength, and function. We also found that those with higher self-efficacy had better muscle function and strength and those with low goal congruence had lower muscle function and strength. These results are consistent with our previous research among CCRC residents which found those with high self-efficacy had numerically greater muscle function and strength [8]. Our study also adds to the literature by demonstrating the importance of goal congruence in the context of physical activity and dietary SMBs and muscle outcomes and that it should be considered in future research.

The results of our significant mediation model suggest that sedentary behavior and MPA partially explain the relationship between self-efficacy and muscle function. These finding support previous research on the role of sedentary behavior and physical activity in improving muscle function [48, 49] and that high levels of self-efficacy are associated with increased engagement in physical activity [50]. These findings together support previous research on the relationship between self-efficacy, physical activity, and muscle function and highlight the importance of addressing both self-efficacy and engagement in physical activity when attempting to improve muscle function [51]. Although these findings support previous research, this relationship has not been previously studied in this way in a population of CCRC residents in the United States.

Our findings suggest implementing multiple health behavior change interventions aimed at promoting eating behaviors and physical activity simultaneously to maximize its potential impact on muscle outcomes. Such interventions should take into consideration the self-management process factors including self-efficacy, goal congruence, aging expectations, and social support. Other determinants such as individual preferences and availability of food and physical activity options are not studied in this research and should be considered when designing future interventions. Such interventions are crucial to promote SMB and muscle outcomes among CCRC residents so they can live independently and avoid transfer to more restricted living environment such as nursing homes.

This study has both strengths and limitations. Inclusion of a variety of factors identified from the Individual and Family Self-Management Theory in the analyses, including the process factors of physical activity and dietary SMBs and muscle outcomes, represents a strength of the analytical approach. Performing objective measure, of physical activity and muscle strength and function is another strength of this study. The study has several limitations worth mentioning. Using a convenience sample limits the generalizability of the findings. The study design was cross-sectional, which does not allow for causal claims. Dietary intake was assessed using FFQ, which might have led to underestimation or overestimation protein and caloric intake. However, the FFQ has good validity and reproducibility among older adults [52]. The newly modified goal congruence scale needs further refinement. Participants were not asked to identify goals but instead were provided with a list of commonly known physical activity and protein intake-related goals. It was not possible, in those with very low scores, to differentiate between having no problem meeting a goal or having no goal in that area. Finally, using the BIS may underestimate fat mass, resulting in artificially high fat-free mass values due to the common problem of dehydration in older adults. However, BIS is inexpensive, noninvasive, and well-correlated with MRI and DXA predictions among older adults [35].

## Conclusions

In conclusion, this study found relationships between the self-management process factors and physical activity and SMBs and muscle outcomes. Specifically, goal congruence predicted engagement in sedentary behavior and light physical activity, self-efficacy and social support predicted moderate physical activity and caloric intake, and self-efficacy and goal congruence predicted muscle function and strength. Sedentary behavior and achieving at least 3 min of MPA also mediated the relationship between self-efficacy and muscle function. Understanding the factors associated with poor SMBs and muscle outcomes can inform the development of interventions as well as matching interventions to CCRC residents. Designing interventions to address modifiable factors associated with poor SMBs and muscle outcomes may prevent sarcopenia, promote independence, and improve quality of life.

## Data Availability

The datasets used and/or analyzed during the current study are available from the corresponding author on reasonable request.

## Abbreviations

SMBs: Self-management behaviors
CCRC: Continuing care retirement community
SPPB: Short physical performance battery
PROMIS: Patient reported outcome measurement information system
ERA: Expectations regarding aging
SIS: Social influence scale
LPA: Light physical activity
MPA: Moderate physical activity
VPA: Vigorous physical activity
FFQ: Food frequency questionnaires
BIS: Bioelectrical impedance spectroscopy
ASMM: Appendicular skeletal muscle mass
OR: Odds ratio
RDA: Recommended dietary allowance

## References

[1] Bruyère O, Beaudart C, Locquet M, Buckinx F, Petermans J, Reginster JY (2016). Sarcopenia as a public health problem. Eur Geriatr Med.

[2] Cruz-Jentoft AJ, Bahat G, Bauer J, Boirie Y, Bruyère O, Cederholm T (2019). Sarcopenia: revised European consensus on definition and diagnosis. Age Ageing.

[3] Deutz NEP, Bauer JM, Barazzoni R, Biolo G, Boirie Y, Bosy-Westphal A (2014). Protein intake and exercise for optimal muscle function with aging: recommendations from the ESPEN expert group. Clin Nutr.

[4] Yang L, Cao C, Kantor ED, Nguyen LH, Zheng X, Park Y (2019). Trends in sedentary behavior among the US population, 2001-2016. J Am Med Assoc.

[5] Keadle SK, McKinnon R, Graubard BI, Troiano RP (2016). Prevalence and trends in physical activity among older adults in the United States: a comparison across three national surveys. Prev Med.

[6] Fulgoni VL (2008). Current protein intake in America: analysis of the National Health and Nutrition Examination Survey, 2003–2004. Am J Clin Nutr.

[7] Gregorio L, Brindisi J, Kleppinger A, Sullivan R, Mangano KM, Bihuniak JD (2014). Adequate dietary protein is associated with better physical performance among post-menopausal women 60-90 years. J Nutr Heal Aging.

[8] Taani MH, Siglinsky E, Kovach CR, Buehring B (2018). Psychosocial factors associated with reduced muscle mass, strength, and function in residential care apartment complex residents. Res Gerontol Nurs.

[9] Ryan P, Sawin KJ (2009). The individual and family self-management theory: background and perspectives on context, process, and outcomes. Nurs Outlook.

[10] Krein SL, Heisler M, Piette JD, Butchart A, Kerr EA (2007). Overcoming the influence of chronic pain on older patients’ difficulty with recommended self-management activities. Gerontologist.

[11] Scott D, Blizzard L, Fell J, Jones G (2012). Prospective study of self-reported pain, radiographic osteoarthritis, sarcopenia progression, and falls risk in community-dwelling older adults. Arthritis Care Res.

[12] Ellis JL, Kovach CR, Fendrich M, Olukotun O, Baldwin VK, Ke W (2019). Factors related to medication self-management in african american older women. Res Gerontol Nurs.

[13] Warren-Findlow J, Seymour RB, Huber LRB (2012). The association between self-efficacy and hypertension self-care activities among African American adults. J Community Health.

[14] Kim SH (2009). Older people’s expectations regarding ageing, health-promoting behaviour and health status. J Adv Nurs.

[15] Yeom HE (2013). Symptoms, aging-stereotyped beliefs, and health-promoting behaviors of older women with and without osteoarthritis. Geriatr Nurs.

[16] Smith Anderson-Bill E, Winett RA, Wojcik JR. Social cognitive determinants of nutrition and physical activity among web-health users enrolling in an online intervention: The influence of social support, self-efficacy, outcome expectations, and self-regulation. J Med Internet Res. 2011;13(1):e28. 10.2196/jmir.1551. 10.2196/jmir.1551 PMC3221350 21441100

[17] Nasreddine ZS, Phillips NA, Bédirian V, Charbonneau S, Whitehead V, Collin I (2005). The Montreal cognitive assessment, MoCA: a brief screening tool for mild cognitive impairment. J Am Geriatr Soc.

[18] Sheikh JI, Yesavage JA (1986). 9/geriatric depression scale (GDS) recent evidence and development of a shorter version. Clin Gerontol.

[19] HealthMeasures. https://www.healthmeasures.net/search-view-measures?task=Search.search. Accessed 12 July 2021.

[20] Cella D, Yount S, Rothrock N, Gershon R, Cook K, Reeve B, et al. The Patient-Reported Outcomes Measurement Information System (PROMIS): progress of an NIH roadmap cooperative group during its first two years. Med Care. 2007;45(5 Suppl 1):S3–S11. 10.1097/01.mlr.0000258615.42478.55. 10.1097/01.mlr.0000258615.42478.55 PMC2829758 17443116

[21] Haas BK, Northam S (2010). Measuring self-efficacy: development of the physical activity assessment inventory. South Online J Nurs Res.

[22] Im EO, Chee W, Lim HJ, Liu Y, Kim HK (2008). Midlife women’s attitudes toward physical activity. J Obstet Gynecol Neonatal Nurs.

[23] Ryan P, Maierle D, Csuka ME, Thomson A, Szabo A (2013). Computer-based intervention to enhance self-management of calcium and vitamin D intake in women. West J Nurs Res.

[24] Sarkisian CA, Hays RD, Berry S, Mangione CM (2002). Development, reliability, and validity of the expectations regarding aging (ERA-38) survey. Gerontologist.

[25] Chogahara M (1999). A multidimensional scale for assessing positive and negative social influences on physical activity in older adults. J Gerontol Ser B Psychol Sci Soc Sci.

[26] Choi L, Liu Z, Matthews CE, Buchowski MS (2011). Validation of accelerometer wear and nonwear time classification algorithm. Med Sci Sports Exerc.

[27] Troiano RP, Berrigan D, Dodd KW, Mâsse LC, Tilert T, Mcdowell M (2008). Physical activity in the United States measured by accelerometer. Med Sci Sports Exerc.

[28] Freedson PS, Melanson E, Sirard J (1998). Calibration of the computer science and applications, Inc accelerometer. Med Sci Sports Exerc.

[29] Matthews CE (2005). Calibration of accelerometer output for adults. Med Sci Sports Exerc.

[30] Hart TL, Swartz AM, Cashin SE, Strath SJ (2011). How many days of monitoring predict physical activity and sedentary behaviour in older adults?. Int J Behav Nutr Phys Act.

[31] Block G, Hartman AM, Naughton D (1990). A reduced dietary questionnaire: development and validation. Epidemiology.

[32] Boucher B, Cotterchio M, Kreiger N, Nadalin V, Block T, Block G (2006). Validity and reliability of the Block98 food-frequency questionnaire in a sample of Canadian women. Public Health Nutr.

[33] Bauer J, Biolo G, Cederholm T, Cesari M, Cruz-Jentoft AJ, Morley JE (2013). Evidence-based recommendations for optimal dietary protein intake in older people: a position paper from the prot-age study group. J Am Med Dir Assoc.

[34] Institute of Medicine (2005). Dietary reference intakes for energy, carbohydrate, Fiber, fat, fatty acids, cholesterol, protein and amino acids.

[35] Sergi G, De Rui M, Veronese N, Bolzetta F, Berton L, Carraro S (2015). Assessing appendicular skeletal muscle mass with bioelectrical impedance analysis in free-living Caucasian older adults. Clin Nutr.

[36] Mathiowetz V (2002). Comparison of Rolyan and Jamar dynamometers for measuring grip strength. Occup Ther Int.

[37] Guralnik JM, Simonsick EM, Ferrucci L, Glynn RJ, Berkman LF, Blazer DG, et al. A short physical performance battery assessing lower extremity function: association with self-reported disability and prediction of mortality and nursing home admission. J Gerontol. 1994;49(2):M85–94. 10.1093/geronj/49.2.M85. 10.1093/geronj/49.2.m85 8126356

[38] Baron RM, Kenny DA (1986). The moderator–mediator variable distinction in social psychological research: conceptual, strategic, and statistical considerations. J Pers Soc Psychol.

[39] Chakravarthy A, Resnick B (2017). Reliability and validity testing of the MotionWatch 8 in older adults. J Nurs Meas.

[40] Regan K, Intzandt B, Swatridge K, Myers A, Roy E, Middleton LE (2016). Changes in physical activity and function with transition to retirement living: a pilot study. Can J Aging.

[41] Paddon-Jones D, Campbell WW, Jacques PF, Kritchevsky SB, Moore LL, Rodriguez NR (2015). Protein and healthy aging. Am J Clin Nutr.

[42] Plotkin A, Taani MH (2020). Factors associated with food intake, nutritional status, and function among nursing home residents with dementia. Geriatr Nurs.

[43] Naseer M, Forssell H, Fagerström C (2016). Malnutrition, functional ability and mortality among older people aged ≥60 years: a 7-year longitudinal study. Eur J Clin Nutr.

[44] Olaya B, Moneta MV, Doménech-Abella J, Miret M, Bayes I, Ayuso-Mateos JL (2018). Mobility difficulties, physical activity, and all-cause mortality risk in a nationally representative sample of older adults. J Gerontol - Ser A Biol Sci Med Sci.

[45] Mitzner TL, McBride SE, Barg-Walkow LH, Rogers WA (2013). Self-management of wellness and illness in an aging population. Rev Hum Factors Ergon.

[46] Stewart TL, Chipperfield JG, Perry RP, Weiner B (2012). Attributing illness to “old age:” consequences of a self-directed stereotype for health and mortality. Psychol Health.

[47] Luger E, Dorner TE, Haider S, Kapan A, Lackinger C, Schindler K (2016). Effects of a home-based and volunteer-administered physical training, nutritional, and social support program on malnutrition and frailty in older persons: a randomized controlled trial. J Am Med Dir Assoc.

[48] Gell NM, Bouldin ED, Rosenberg D (2019). Time spent in sedentary behavior domains and physical function in u.s. older adults. Innov Aging.

[49] Stenholm S, Koster A, Valkeinen H, Patel KV, Bandinelli S, Guralnik JM (2016). Association of physical activity history with physical function and mortality in old age. J Gerontol - Ser A Biol Sci Med Sci.

[50] Koeneman MA, Verheijden MW, Chinapaw MJM, Hopman-Rock M (2011). Determinants of physical activity and exercise in healthy older adults: a systematic review. Int J Behav Nutr Phys Act.

[51] Brady AO, Straight CR, Evans EM (2014). Body composition, muscle capacity, and physical function in older adults: an integrated conceptual model. J Aging Phys Act.

[52] Mirmiran P, Hosseini Esfahani F, Mehrabi Y, Hedayati M, Azizi F (2010). Reliability and relative validity of an FFQ for nutrients in the Tehran lipid and glucose study. Public Health Nutr.

